# Liraglutide Activates the Nrf2/HO-1 Antioxidant Pathway and Protects Brain Nerve Cells against Cerebral Ischemia in Diabetic Rats

**DOI:** 10.1155/2018/3094504

**Published:** 2018-02-12

**Authors:** Caihong Deng, Jun Cao, Jiangquan Han, Jianguo Li, Zhaohun Li, Ninghua Shi, Jing He

**Affiliations:** ^1^Department of Neurology, The Fifth Affiliated Hospital of Zunyi Medical College, Zhuhai 519100, China; ^2^Department of Pharmacy, Xindu District People's Hospital of Chengdu, Chengdu 610500, China; ^3^Department of Emergency, The Fifth Affiliated Hospital of Zunyi Medical College, Zhuhai 519100, China; ^4^Department of Neurology, Zhuhai People's Hospital, Zhuhai 519100, China

## Abstract

This study aimed to determine the effect of liraglutide pretreatment and to elucidate the mechanism of nuclear factor erythroid 2-related factor (Nrf2)/heme oxygenase-1 (HO-1) signaling after focal cerebral ischemia injury in diabetic rats model. Adult male Sprague-Dawley rats were randomly divided into the sham-operated (S) group, diabetes mellitus ischemia (DM + MCAO) group, liraglutide pretreatment normal blood glucose ischemia (NDM+MCAO+L) group, and liraglutide pretreatment diabetes ischemia (DM + MCAO + L) group. At 48 h after middle cerebral artery occlusion (MCAO), neurological deficits and infarct volume of brain were measured. Oxidative stress brain tissue was determined by superoxide dismutase (SOD) and myeloperoxidase (MPO) activities. The expression levels of Nrf2 and HO-1 of brain tissue were analyzed by western blotting. In the DM + MCAO + L group, neurological deficits scores and cerebral infarct volume seemed to decrease at 48 h after MCAO cerebral ischemia compared with those in DM + MCAO group (*P* < 0.05). In addition, the expression of Nrf2 and HO-1 increased in 48 h at liraglutide pretreatment groups after MCAO cerebral ischemia if compared with those in the DM + MCAO group (*P* < 0.05). Furthermore, the DM + MCAO + L group has no significant difference compared with the NDM + MCAO + L group (*P* > 0.05). To sum up, alleviating effects of liraglutide on diabetes complicated with cerebral ischemia injury rats would be related to Nrf2/HO-1 signaling pathway.

## 1. Introduction

Ischemic stroke is characterized by high rates of morbidity, mortality, disability, and relapse [1, 2]. At present, intravascular thrombolysis and embolectomy are achieved via the blood vessels, while other therapeutic means remain unsatisfactory. Diabetes mellitus (DM) is an important risk factor for cerebral infarction. Compared with nondiabetic patients, the incidence of cerebral infarction in patients with diabetes is 1.8–6.0 times higher [3]. Liraglutide is a long-acting synthetic analog of glucagon-like peptide-1 (GLP-1) in the treatment of type 2 diabetes drugs and has been sold in the United States, Europe, and China. It is chemically similar to natural GLP-1, with 97% homology [4], and also has some biological effects of GLP-1. In 2013, Sato et al. suggested that liraglutide reduced neuronal damage caused by cerebral ischemia in diabetic and nondiabetic rats through antioxidative stress and antiapoptosis pathways [5]. Previous studies have confirmed that the Nrf2/HO-1 signaling pathway plays an important role in antioxidative stress. In view of liraglutide's antioxidative stress activity, we speculated that it could activate the nuclear factor erythroid 2-related factor (Nrf2)/heme oxygenase-1 (HO-1) pathway in the treatment of diabetes complicated with cerebral infarction. We recommend explaining the relationship between Nrf2/HO-1 signaling pathway and neuronal damage caused by cerebral ischemia. This could make your investigation more convincing. Generally speaking, introduction is too simple and therefore needs more information inserted into this part.

## 2. Materials and Methods

### 2.1. Animals

Adult male Sprague-Dawley rats (280–320 g) were purchased from Guangdong Provincial Medical Laboratory Animal Center. Animals were housed in cages at constant temperature (22°C) and relative humidity (55%) with a 12-h light–12-h dark cycle (light 06.00–18.00 h). The experimental protocol was approved by the Animal Ethics Committee of Guangdong Medical Laboratory Animal Center and performed in accordance with the National Institutes of Health Guide for the Care and Use of Laboratory Animals. Precautions were taken to minimize suffering and the number of animals used in each experiment. Rats were randomly divided into four groups with 12 rats per group: sham-operated (S) group, liraglutide pretreatment normal blood glucose cerebral ischemia (NDM + MCAO + L) group, liraglutide pretreatment diabetes mellitus cerebral ischemia (DM + MCAO + L) group, and diabetes mellitus cerebral ischemia (DM + MCAO) group.

### 2.2. Type 1 Diabetes Mellitus Model

A rat model of type 1 DM was established by intraperitoneal (i.p.) injection of streptozotocin (STZ; Sigma, USA) at 20 mg/kg in the first 24 h and at 35 mg/kg on the next day following 8 h of fasting [6]. The animals were allowed normal drinking water and feeding for 7 days, and there was no blood glucose intervention until on the diabetes diet control on 7 consecutive days. The changes in symptoms and signs in rats were observed. A blood glucose meter (Roche, Germany) was used to determine blood glucose in a tail vein sample. The diabetes model was deemed successful when random blood glucose was more than 13.9 mm/L and accompanied by polydipsia, polyuria, and weight loss. Rats were excluded for not up to 13.9 mm/L of blood glucose or because of death before the start of the experiment.

### 2.3. Middle Cerebral Artery Occlusion Model

After DM induction, middle cerebral artery occlusion (MCAO) models were established once blood glucose levels remained stable for 7–14 days. Rats were anesthetized with chloral hydrate (300 mg/kg, i.p.) and subjected to MCAO as previously described with slight modifications [7]. Briefly, the left common carotid artery, internal carotid artery (ICA), and external carotid artery (ECA) were exposed, and the ECA was dissected distally. A special nylon suture with a rounded tip (line diameter of 0.28 mm, head diameter of 0.36 ± 0.02 mm) was inserted into the ICA through the ECA stump and was gently advanced to occlude the middle cerebral artery (MCA). The animals that died after ischemia induction or that had evidence of subarachnoid hemorrhage on extraction of brain tissue were excluded. Skin and subcutaneous tissue were sutured immediately after the MCAO. Body temperature was monitored with a rectal probe and maintained at 37°C during the entire procedure. Rats in the sham group were manipulated in the same way but without MCAO.

### 2.4. Liraglutide Administration

Liraglutide (Novo Nordisk Denmark) was dissolved in saline and administered at a dose of 100 *μ*g/kg (for the two groups of liraglutide pretreatment). The studies were only performed in the 100 *μ*g/kg liraglutide group, because this dose given twice daily in rats can be converted to the human equivalent dose of 1.92 mg/day, assuming a 60-kg human, according to FDA^S^ guidance [8] (i.p.) for 7 days prior to MCAO [9]. The remaining drug was stored in a refrigerator at 4°C. The rats in the sham group with normal blood glucose and ischemia group with normal blood glucose were injected with sterile sodium citrate buffer. The diabetic cerebral ischemia liraglutide group and normal blood glucose cerebral ischemia liraglutide group were given a lower left abdominal cavity injection of liraglutide at 100 *μ*g/kg, every 12 h for 7 days after MCAO. The rats in the sham group with normal blood glucose and diabetic cerebral ischemia group were injected with an equal volume of normal saline.

### 2.5. Neurological Deficit Scores and Determination of Cerebral Infarct Volume

Neurological functions were evaluated according to 5-point scoring criterion of Longa et al. [7]: 0 points, no neurological deficit; 1 point, failure to extend left forepaw fully; 2 points, circling to the left; 3 points, falling to the left; and 4 points, no walking spontaneously and having a depressed level of consciousness. The first evaluation was performed 3 h after the operation, and animals that were given a score of 0 or 4 were excluded (except the sham operation group). Neurological function was reappraised at 24 and 48 h after the MCAO.

Infarct volume was examined 48 h after MCAO; the rats were anesthetized with chloral hydrate (10%, stored in 4°C refrigerator) and decapitated. The brains were quickly removed and placed in cold saline for 5 min. The tissues were cut at 2-mm intervals from the frontal pole to obtain five coronal sections that were stained with 2% 2,3,5-triphenyltetrazolium chloride (TTC; Sigma, USA) for 30 min followed by overnight immersion in 10% formalin. The infarcted regions were quantified by assessing each section with Image J software (National Institutes of Health, Bethesda, Maryland, USA). The infarct size as a percentage of the whole brain was calculated [10].

### 2.6. Measurement of Superoxide Dismutase (SOD) and Myeloperoxidase (MPO) Activity in Brain Tissue Homogenate

The rats were sacrificed at 48 h after MCAO and brains were rapidly removed. Brain tissue samples (about 100 mg) were obtained, and a homogenate (10%) was prepared by homogenizing the sample in normal saline at 1 : 9 ratio, and the supernatant was obtained by refrigerated centrifugation (10 min, 3000 rpm) to measure superoxide dismutase (SOD) and myeloperoxidase (MPO) activity. SOD activity in the brain tissue was determined according to the method described by Kakkar et al. [11]. A xanthine and xanthine oxidase system was used to generate superoxide radicals, which reacted with *p*-iodonitrotetrazolium violet (INT), converting it into a red formazan product. Spectrometry was used to measure absorbance of the sample at 550 nm. MPO activity in the brain tissue was determined according to the method previously described [12]. Briefly, MPO activity was measured by the hydrogen peroxide reduction method.

SOD and MPO activities were expressed as U/mg protein. The SOD kit (A001-1) and MPO kit were obtained from Nanjing Jiancheng Bioengineering Institute (Nanjing Jiancheng, China).

### 2.7. Measurement of Nrf2 and HO-1 Protein Expression in Brain Tissue

At 48 h after MCAO, the rats (*n* = 6 per group) were anesthetized with chloral hydrate (10%, stored in 4°C refrigerator) and decapitated. The brains were quickly removed and stored in a −80°C freezer. Total proteins in the cerebral cortex samples were extracted as previously described. Nuclear proteins in the cortical tissues were isolated using RIPA lysis buffer and Cytoplasmic Extraction Reagents (Boster Company, Wuhan, China) according to the manufacturer's instructions. Protein concentrations were determined by using the bicinchoninic acid protein assay with bovine serum albumin as the standard [13] (McConkey, 1984). Protein expression was detected as follows. In brief, the protein samples (20 mg) were separated on 10% sodium dodecyl sulfate-polyacrylamide gels and then transferred to a nitrocellulose membrane. The membrane was blocked with 5% nonfat dry milk in Tris-buffered saline with Tween-20 (TBST) and incubated overnight at 4°C with rabbit anti-rat Nrf2 monoclonal antibody (1 : 1000; Abcam Company, USA) and rabbit anti-rat HO-1 monoclonal antibody (1 : 2000; Abcam Company). Rabbit anti-rat *β*-actin monoclonal antibody (1 : 5000; Boster Company, Wuhan, China) was used for the internal control. After extensive rinsing with TBST, the membranes were incubated with goat anti-rabbit IgG conjugated with horseradish peroxidase (1 : 8000; Boster Company, Wuhan, China) for 1 h at 37°C. Bound antibody was detected by using an enhanced chemiluminescence detection system (ECL, Boster Company, Wuhan, China) and exposure of membranes to X-ray films. The ratio of optical density value as protein expression in each sample was analyzed with Image J software.

### 2.8. Statistical Analysis

SPSS 13.0 software was used for statistical analysis. The results were expressed as mean ± SEM for normally distributed data. One-way analysis of variance (ANOVA) was used to analyze the difference between multiple means. The LSD *t*-test was used to analyze the difference between two groups. *P* < 0.05 was considered statistically significant.

## 3. Results

### 3.1. Blood Glucose Measurement

**Figure 1:**
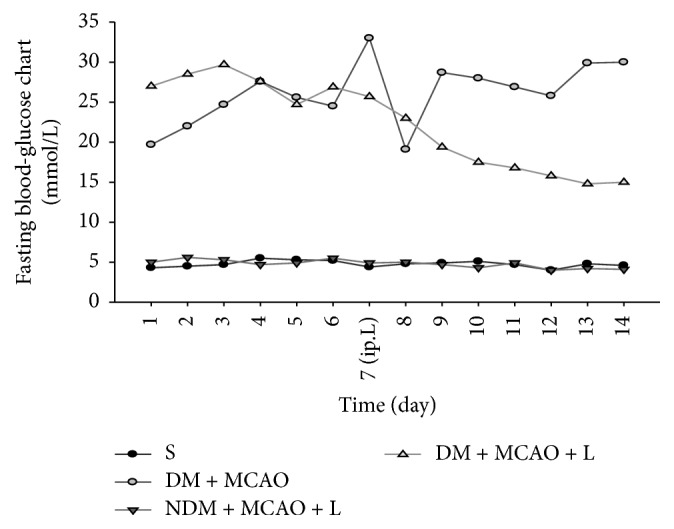
Fasting blood glucose concentrations. There was no change in blood glucose in the sham group and blood glucose in the NDM + MCAO + L. DM + MCAO and DM + MCAO + L groups increased significantly (*P* < 0.05); after liraglutide administration, the blood glucose level in the DM + MCAO + L group decreased significantly when compared with that in DM + MCAO group (*P* < 0.05). Values are mean ± SD, *n* = 12, all *P* < 0.05.

In 24 h after the diabetes model was established, tail vein blood glucose of each rat was monitored regularly for 14 days. Blood glucose in the DM + MCAO and DM + MCAO + L groups increased significantly compared with the S group (*P* < 0.05). In addition, rats treated with liraglutide reduced blood glucose levels in DM + MCAO + L groups (*P* < 0.05) (Figure 1). These results indicated the diabetes model was deemed successful and the liraglutide reduced blood glucose levels.

### 3.2. Neurological Deficit Evaluation

**Figure 2:**
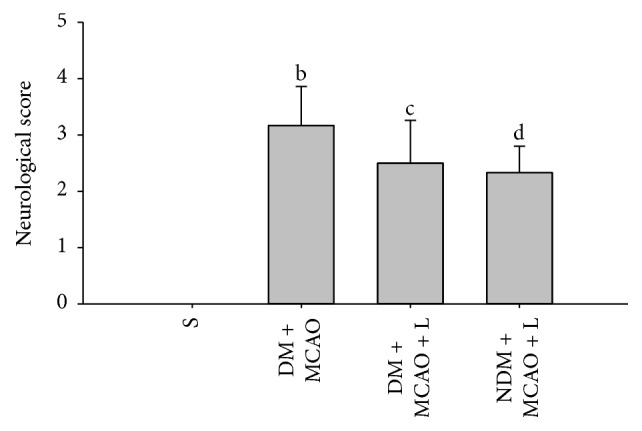
Neurological deficit score in each group. In the sham operation group, the neurological deficit score was 0. The neurological deficit score in the DM + MCAO + L group was significantly lower than in the DM + MCAO group (*bc*  *P* < 0.05); DM + MCAO + L and NDM + MCAO + L groups did not differ significantly in neurological deficit score (*cd*  *P* > 0.05). Values are mean ± SD, *n* = 12, all *P* < 0.05.

The neurological deficit score showed that there was no neurological deficit in the sham operation group (score 0). Liraglutide decreased the neurological deficit score in the DM + MCAO + L and NDM + MCAO + L groups (*P* < 0.05) (Figure 2). The DM + MCAO + L and NDM + MCAO + L groups have no significant difference in neurological deficit score (*P* > 0.05) (Figure 2). The result indicated that the liraglutide improved neurological deficit score in rats after MCAO.

### 3.3. Cerebral Infarction Volume

**Figure 3:**
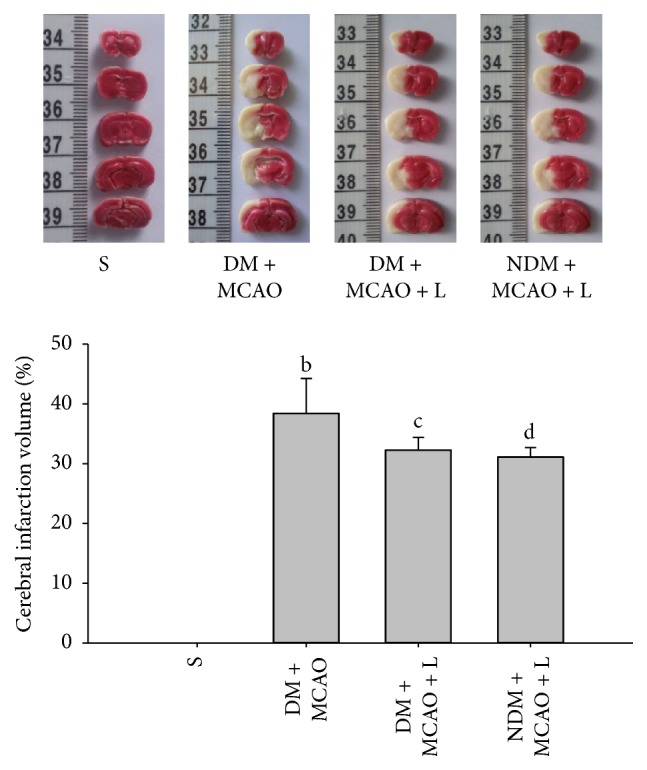
Cerebral infarction volume in each group. There was no cerebral infarction in the S group; the infarction volume in the S group was significantly lower than in the other cerebral ischemia group (*P* < 0.05). The infarction volume in the DM + MCAO + L group was significantly lower than in the DM + MCAO group (*cb*  *P* < 0.05). The DM + MCAO + L and NDM + MCAO + L groups did not differ significantly in infarction volume (*cd*  *P* > 0.05). Values are mean ± SD, *n* = 6, all *P* < 0.05.

TTC staining showed that no infarct was observed in sham operation group. Liraglutide decreased infarct volume in the DM + MCAO + L and NDM + MCAO + L groups (*P* < 0.05) (Figure 3). But comparing the DM + MCAO + L group and NDM + MCAO + L group, there was no significant difference (*P* > 0.05) (Figure 3). The result indicated that the liraglutide attenuated cerebral infarction volume in diabetic rats after MCAO.

### 3.4. Determination of SOD and MPO Activity

**Figure 4:**
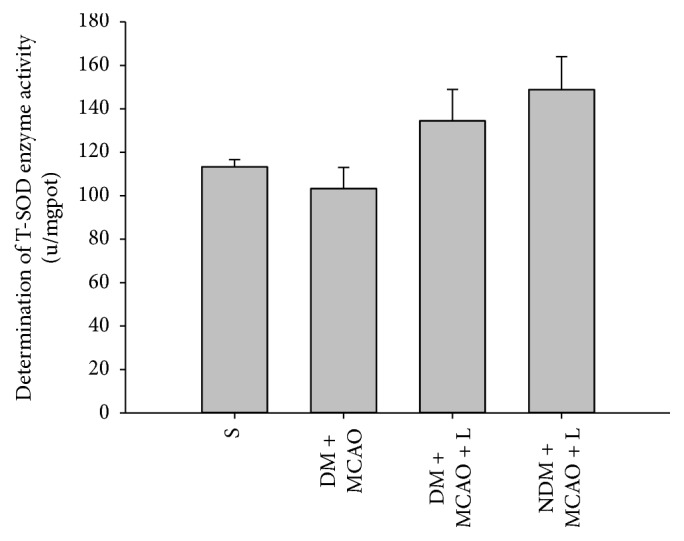
SOD activity in each group. There are significant differences in SOD activity levels between the S, DM + MCAO, NDM + MCAO + L, and DM + MCAO + L groups (*P* < 0.01); SOD activity in the NDM + MCAO + L and DM + MCAO + L groups is significantly higher than in the DM + MCAO group (*P* < 0.05). SOD activity levels in the NDM + MCAO + L and DM + MCAO + L groups did not differ significantly (*P* > 0.05). Values are mean ± SD, *n* = 6, all *P* < 0.05.

**Figure 5:**
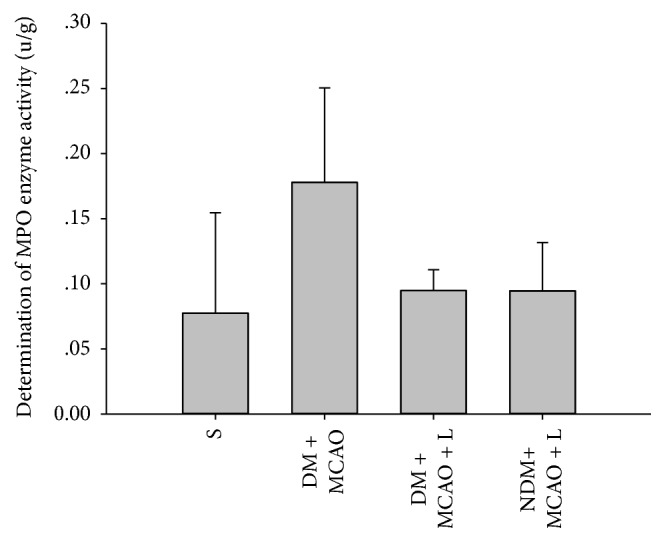
MPO activity in each group. There were significant differences in MPO activity levels in the S, DM + MCAO, NDM + MCAO + L, and DM + MCAO + L groups (*P* < 0.01). MPO activity in the NDM + MCAO + L and DM + MCAO + L groups is significantly higher than in the DM + MCAO group (*cdb*  *P* < 0.05). MPO activity levels in the NDM + MCAO + L and DM + MCAO + L groups have no significant difference (*cd*  *P* > 0.05). Values are mean ± SD, *n* = 6, all *P* < 0.05.

After the injection of liraglutide the results showed differences in experimental groups respectively (*P* < 0.01). Liraglutide increased SOD and decreased MPO activity levels in the DM + MCAO + L and NDM + MCAO + L groups (*P* < 0.05) (Figures 4 and 5). But the NDM + MCAO + L and DM + MCAO + L groups have no significant difference in SOD and MPO activity (*P* > 0.05) (Figures 4 and 5). The result indicated that liraglutide increases antioxidant SOD and decreases inflammatory substance MPO in diabetic rats after MCAO.

### 3.5. Western Blot

**Figure 6:**
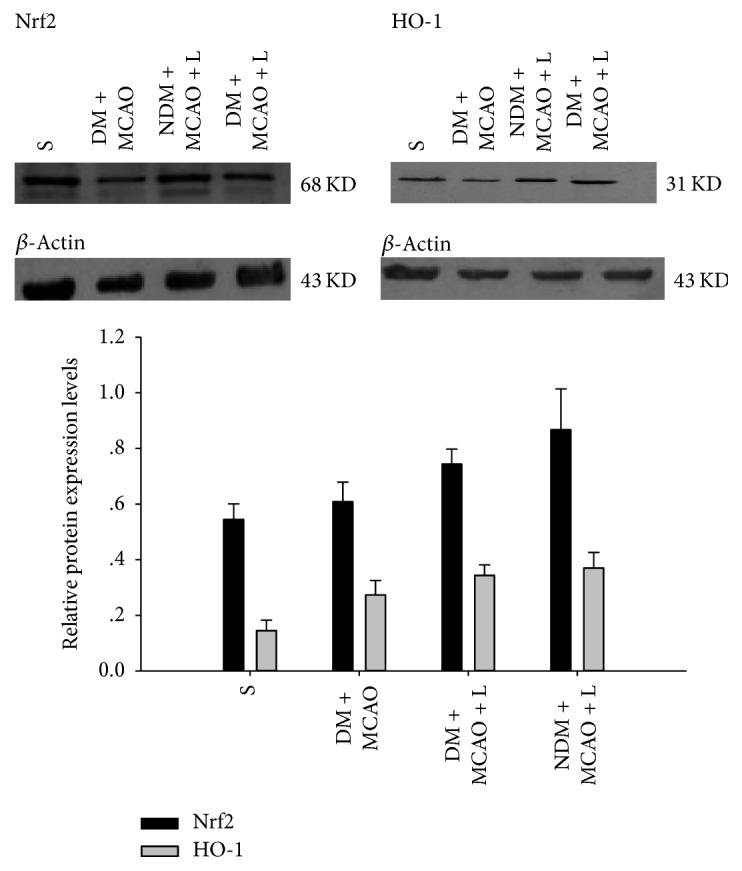
Western blot measurement of Nrf2 and HO-1 protein expression. There are significant differences in the expression levels of Nrf2 and HO-1 between the sham, DM + MCAO, NDM + MCAO + L, and DM + MCAO + L groups (*P* < 0.01). Nrf2 and HO-1 expression in the NDM + MCAO + L and DM + MCAO + L groups is significantly higher than in the DM + MCAO group (*P* < 0.05), but the NDM + MCAO + L and DM + MCAO + L groups have no significant difference in Nrf2 and HO expression levels (*P* > 0.05). Values are mean ± SD, *n* = 6, all *P* < 0.05.

Western blot results show that there are significant differences in the expression levels of Nrf2 and HO-1 in experimental groups respectively (*P* < 0.01) (Figure 6). Liraglutide decreased the expression levels of Nrf2 and HO-1 in the NDM + MCAO + L and DM + MCAO + L groups (*P* < 0.05) (Figure 6). But the NDM + MCAO + L and DM + MCAO + L groups have no significant difference in expression levels of Nrf2 and HO-1 (*P* > 0.05) (Figure 6). The result indicated that Nrf2 and HO-1 expression were upregulated in diabetic rats after MCAO and the liraglutide injection augmented expression of Nrf2 and HO-1 in the cerebral ischemia tissue.

## 4. Discussion

Diabetes is a chronic disease and a serious threat to human health. It is often complicated with cardiac and cerebral vascular diseases. Previous studies have shown that DM is an independent risk factor for cerebral infarction and/or poor prognosis [14]. This study demonstrates that, in the diabetic rats with cerebral ischemia, neurological symptoms are more serious than in other groups. Data in current investigation indicated that neurological deficit scores in diabetic rats with cerebral ischemia were higher compared with those in other groups. We also found that the infarction area in rats with DM and ischemia was significantly greater than in normal blood glucose rats with ischemia. These findings suggest that DM is one of the important factors leading to cerebral ischemia exacerbation.

In recent years, more and more studies have proved that oxidative stress is one of the important factors in diabetes that aggravate the damage in ischemic brain tissue [15, 16]. It has been shown that, in diabetes, the systemic and local inflammatory reaction is enhanced and the levels of serum inflammatory markers are increased. Regardless of the duration of DM, oxidative stress and the expression of antioxidative stress factors are observed in various tissues of patients. Therefore, it is suggested that diabetes is associated with cerebral ischemia and oxidative stress [17].

Nrf2 is one of the most important nuclear transcription factors in eukaryotic cells. When the occurrence of diabetes complicated with cerebral infarction, the Nrf2/HO-1 signaling pathway is activated and regulates other antioxidant enzymes and phase II detoxifying enzymes. These enzymes can be against the damage of nerve cells.

Previous studies have shown that overexpression of Nrf2 can significantly reduce ischemic brain injury [17]. It has been confirmed that there is oxidative stress injury in ischemic neuronal cells immediately after the cerebral ischemia occurs and that the mechanism of antioxidative stress is activated. The expression of nuclear transcription factor Nrf2 and downstream HO-1 was significantly increased. Many studies have shown that when cerebral ischemia occurs, Nrf2 is activated in neuronal cells, vascular endothelial cells, and glial cells [18]. Meanwhile, it prompts the expression of the downstream of antioxidant proteasome, phase II detoxifying enzymes, and HO-1 protein. Thus, Nrf2 is considered to be one of the initiating mechanisms of cerebral ischemia and oxidative stress. Tu et al. found that, in a permanent cerebral ischemia mouse model, exposure to 20 mmol/L tBHQ for 6 hours after ischemia significantly decreased infarct size [19], and at the same time, Nrf2 and HO-1 expression increased. However, this phenomenon cannot be found in Nrf2 knockout mice. Jiang et al. showed that oxymatrine protected neurons after cerebral ischemia/reperfusion injury by activating the Nrf2/HO-1 signaling pathway [20]. Oxymatrine increased Nrf2 and HO-1 expression in the cerebral cortex, after 6 h in the cerebral ischemia/reperfusion model, which peaked at 48 h, while ischemic infarction and edema were significantly reduced. It had a clear protective effect in ischemic nerve cells.

Our study shows that the DM with cerebral ischemia group has significantly increased expression of Nrf2 and HO-1 compared to the sham operation group. The expressions of Nrf2 and HO-1 are involved in the process of occurrence and development of diabetes complicated with cerebral ischemia. In addition, the expression of Nrf2 and HO-1 is significantly lower in DM with cerebral ischemia group than in the normal blood glucose with cerebral ischemia group, which suggests that diabetes can aggravate the injury to ischemic cells. It is also one of the important mechanisms of diabetic cerebral ischemia injury.

### 4.1. Nrf2 Can Regulate the Expression of HO-1 Protein

The oxidative/antioxidant system is activated when diabetes complicated with cerebral ischemia occurs. The oxidation of substances increases substantially in cells, producing large amounts of oxygen free radicals, and increased SOD activity may be a direct response to the extent of oxygen free radical scavenging [21]. After the occurrence of diabetes complicated with cerebral ischemia, the inflammatory reaction is considered another important factor in aggravating brain cell injury. MPO is an important factor in the inflammatory response, the determination of the increase of MPO can be confirmed to be one of the mechanisms of cerebral ischemia aggravated diabetes [22].

The results show that SOD and MPO are significantly higher in the diabetes complicated with cerebral ischemia group than in the sham operation group, and it is confirmed that DM is one of the most important factors in cerebral ischemia injury. The liraglutide pretreatment diabetes ischemia group of SOD and MPO enzyme increases significantly compared to the diabetes complicated with cerebral ischemia group. It can explain that the liraglutide has anti-inflammatory and protective effect of ischemic brain cells.

Many past studies have demonstrated that liraglutide has an antioxidative effect [23]. It can protect nerve cells from cerebral ischemic injury. It has a clear protective effect on injured myocardial cells, islet cells, nerve cells, and so on.

There had been no earlier studies showing a neuroprotective effect of liraglutide in diabetic rats with cerebral ischemia injury. This study demonstrates that liraglutide has a protective effect on brain nerve cells in rats with diabetes mellitus combined with cerebral ischemic neuronal injury. The western blot findings for Nrf2 and HO-1 expression levels have showed that the protective effect of liraglutide on brain nerve cells in diabetes complicated with cerebral ischemia injury may be through the activation of the Nrf2/HO-1 signaling pathway.

Further* in vitro* studies are needed to elucidate the molecular mechanism of liraglutide against diabetic cerebral ischemia.

## 5. Conclusion

Liraglutide's alleviating effects on diabetes complicated with cerebral ischemia injury rats may be related to upregulation of Nrf2/HO-1 protein expression. Our findings have shed light on the effects of liraglutide, and further study is necessary to determine its mechanism* in vitro*.
